# A Retrospective Cohort Study to Determine Whether the Previous Route of Delivery Affects the Uterine Artery Blood Flow

**DOI:** 10.7759/cureus.39552

**Published:** 2023-05-26

**Authors:** Elif G Aygun, Emine Karabuk, Talat Umut Kutlu Dilek

**Affiliations:** 1 Obstetrics and Gynecology, Acibadem Atakent University Hospital, Istanbul, TUR; 2 Obstetrics and Gynecology, Acibadem University, Istanbul, TUR; 3 Perinatology, Baskent University Faculty of Medicine, Istanbul, TUR

**Keywords:** perfusion index ratio, doppler us, umbilical artery, uterine artery doppler, cesarean section

## Abstract

Introduction: In our study, we aimed to investigate the effect of the previous delivery route on uterine artery pulsatility index (PI) and obstetric outcome. We aim to assess the effects of previous cesarean delivery (CD) and placental location on first- and second-trimester uterine artery Doppler indices as well as first-trimester pregnancy-associated plasma protein-A (PAPP-A) multiples of the median (MoM) levels in the subsequent pregnancy.

Materials and methods: We designed a retrospective cohort study to collect the participants’ clinical and uterine artery Doppler. Data regarding pregnant women’s first- and second-trimester exams, who were referred to our maternal-fetal medicine unit, were collected from hospital records between June 2015 and December 2019.

Results: Uterine artery PI MoM values were not different between the cases with the anterior and non-anterior placental locations. No significant difference was found in the first- and second-trimester uterine artery PI MoM values by delivery route (p = 0.57). However, the intrauterine growth restriction rate was higher in the CD group (p < 0.001).

Conclusion: In this study, we compared the uterine blood flow indices between the previous cesarean and vaginal delivery groups. We observed no significant difference between the patients with different delivery routes.

## Introduction

Cesarean delivery (CD) rates are increasing progressively all over the world [1]. Many studies support increased adverse maternal and neonatal outcomes in future pregnancies [1]. History of previous CD has been reported with increased risk for intrauterine demise, preterm labor or delivery, low birth weight, preeclampsia, malpresentation, placental invasion anomalies, and uterine rupture. The mechanism behind these adverse effects is not fully understood. Some studies suggest that CD results in uterine tissue devascularization and scar tissue, resulting in a weak trophoblastic invasion, abnormal uteroplacental blood flow, placental invasion anomalies, and a decline in placental function in subsequent pregnancies [2,3-7].

Increased uterine artery pulsatility index (PI), resistance index (RI), and persistent uterine artery diastolic notch are valuable markers for impaired uteroplacental blood flow. PI is dependent on the trophoblastic invasion of the uterine myometrium. Parity may affect uterine artery PI values by cardiovascular remodeling after delivery [8]. A limited number of studies in the literature investigate the effect of previous CD on uterine artery indexes. Those studies indicate that previous CD might be linked with decreased uterine artery blood flow and increased uterine artery PI and RI values [4-6].

We aim to assess the effects of previous CD and placental location on first- and second-trimester uterine artery Doppler indices in the subsequent pregnancy.

This article was previously presented as an abstract at the 2022 TAJEV Annual Scientific Meeting on May 29, 2022.

## Materials and methods

This retrospective cohort study was conducted among pregnant women who were referred to our maternal-fetal medicine unit for the first-trimester anatomic survey and aneuploidy screening between June 2015 and December 2019. This study was approved by the local ethics committee of our institution, Acibadem University Medical Research Ethical Committee (approval number: 2019-11/21). Multiplets, fetuses with structural anomalies or aneuploidies, pregnant women who have a history of more than one delivery (including grand multiparas), pregnancy by assisted reproductive techniques (ART), previous uterine surgery other than cesarean section (CS), acetylsalicylic acid, low molecular weight heparin use during pregnancy, and maternal history of chronic hypertension, antiphospholipid syndrome, systemic lupus erythematosus (SLE), and overt diabetes were excluded from the study.

Our original cohort was composed of 1,500 cases in total. After excluding patients who did not meet the inclusion criteria and those with incomplete clinical records (including pregnancy outcome, delivery time, delivery route, lost during follow-up, and delivered in another medical center), a total of 228 cases remained for the statistical analysis.

Data collection

Clinical data and ultrasound measurements of participants were reviewed from the hospital records. Ultrasound measurements were collected from the long-term storage and ViewPoint 6.0 software (General Electric, Milwaukee, United States). Demographic data including age, number of previous pregnancies, deliveries, delivery route, smoking, hypertension, diabetes, treatment, smoking history, past medical history, pregnancy outcome (growth restriction, preeclampsia, gestational diabetes, oligohydramnios, premature rupture of membranes, and preterm delivery), birth weight, and gestational age at delivery were collected from the hospital data of the individual patient. Also, pregnancy-associated plasma protein-A (PAPP-A) MoM values, body mass index at the first-trimester exam, and mean arterial blood pressure (mmHg) between the 11th and 14th weeks of gestation were recorded. All ultrasound exams were performed by Voluson E8 Expert equipped with RMC 6 MHz convex matrix array transducer (General Electric Healthcare, Milwaukee, WI, USA) and E10 equipped with C4-8 MHz convex array transducer (General Electric Healthcare, Milwaukee, WI, USA). All ultrasound exams were performed by a single operator (maternal-fetal medicine specialist). Uterine artery Doppler exam is performed routinely in our unit at 11-14 weeks and 18-23 weeks.

Ultrasound technique

Uterine artery Doppler examination was performed by transabdominal technique at 11-14 weeks and 18-23 weeks of gestation. The transabdominal transducer obtained the sagittal section of the cervix; following the detection of the cervical canal, the transducer was moved both laterally on both the right and left sides to find uterine arteries by color flow mapping. When the paracervical vascular plexus was seen, the uterine artery was identified by color Doppler imaging at the cervix-uterus junction. Uterine artery flow was measured at this region before branching to the arcuate arteries. The color box was narrowed, and the velocity scale and filter were adjusted. The Doppler gate was set to 2 mm to cover the whole vessel, and the angle of insonation was less than 30°. The target for the PI measurement was the ascending branch of the uterine artery at the point closest to the internal OS (cervical outlet of the uterus) [9].

In the second-trimester exams, the transducer was placed on the lower quadrant of the abdomen and moved medially to find the uterine artery, the apparent crossover with the external iliac artery. Measurements were taken approximately 1 cm distal to the crossover point. The Doppler gate was adjusted to cover the whole vessel, and the insonation angle was less than 30°. Doppler signal was updated until three consecutive waveforms had been obtained. The PI of both uterine arteries was measured, and the mean PI was calculated. It was recorded in an early diastolic notch in the uterine artery. Diastolic notch was defined as a persistent decrease in blood flow velocity in early diastole below the peak diastolic velocity. Uterine artery PIs were considered abnormal if the values were greater than the 95th percentile. Also, multiples of the median (MoM) for uterine artery were calculated for each patient using the following formula [4]:

$PI\ MoM\ =\ \frac{PI\ values\ of\ the\ patient}{Median\ PI\ values\ based\ on\ the\ gestational\ age}$

MoM values of uterine artery PIs were calculated for both exams. In addition, patients' follow-up data were reviewed from the hospital database for the oligohydramnios (defined as single deepest vertical pocket amniotic fluid ≤ 2 cm excluding premature rupture of membranes), placental abruption (separation of the placenta before the delivery of a fetus with a clinical feature of vaginal bleeding, severe sudden onset pain, and uterine tenderness, after 20th week of gestation), intrauterine growth restriction (defined as estimated fetal weight by ultrasound less than the 10th percentile or <2 standard deviations), hypertensive disorders of pregnancy (defined as new-onset hypertension, i.e., systolic blood pressure [BP] > 140 mmHg and/or diastolic BP > 90 mmHg in at least two occasions four hours apart after the 20th week of gestation). Also, proteinuria ≥1 + on a random urine sample or >300 mg in 24-hour urine collection with hypertension classified as preeclampsia, gestational age at delivery, intrauterine fetal death (fetal demise after 20 weeks of gestation), and birth weight at delivery and delivery route are all considered.

Statistical analysis

All statistical analysis was performed by MedCalc statistical analysis software, version 11 (MedCalc Software Ltd., Ostend, Belgium). The Kolmogorov-Smirnov test estimated the distribution of data. Normally distributed data were expressed as mean ± standard deviation (SD). Median and quartiles were used for non-normally distributed data. Student’s t-test and analysis of variance (ANOVA) were used for the comparison of normally distributed data. The Mann-Whitney U and Kruskal-Wallis tests are used for the comparison of variables that do not fit the normal distribution. Finally, the Chi-square test between the groups compared categorical data. A p-value of <0.05 was considered statistically significant.

## Results

Our original cohort was composed of a total of 1500 cases. After the exclusion of cases that did not meet the inclusion criteria and incomplete clinical records (including pregnancy, outcome, delivery time, route, lost during follow-up, and delivered in the other medical center), a total of 228 cases remained for the statistical analysis. Therefore, these 228 cases with a history of previous delivery were classified into the vaginal delivery group (Group 1, n = 102 cases) and the CD group (Group 2, n = 126 cases). A total of 108 cases (47.3%) had anteriorly located placenta, and 101 (44.3%) cases had posteriorly located placenta. The remaining 19 (8.33%) cases had fundal or lateral placental locations. Demographic and clinical data are summarized in Table 1.

**Table 1:** Demographic and clinical data of vaginal and cesarean delivery groups *p < 0.05 was statistically significant; variables except age are expressed as a median. BMI: Body mass index; PAPP-A: Pregnancy-associated plasma protein-A; PI: Pulsatility index; MoM: Multiples of the median.

	Vaginal delivery (n = 102)	Cesarean delivery (n = 126)	p-values
Age (years)	31.71 ± 4.14	33.31 ± 4.14	0.006*
BMI (kg/m^2^)	24.15 (16.85-33.98)	24.84 (18.29-37.28)	0.37
First-trimester exam week	12.4 (11-14.6)	12.4 (11.1-14.5)	0.65
Second-trimester exam week	21.1 (18.2-24)	20.6 (18-23.5)	0.23
PAPP-A	1.29 (0.31-8.1)	1.19 (0.27-3.6)	0.44
Mean arterial pressure (mmHg)	73 (60-96.6)	73.3 (60-110)	0.25
Uterine artery PI MoM in the first trimester	0.8473 (0.35-1.39)	0.8 (0.3-1.72)	0.299
Uterine artery PI MoM in the second trimester	0.89 (0.5-2.1)	0.83 (0.6-1.56)	0.13
Gestational age at delivery	39 (23-41)	39 (19-41)	0.16
Birthweight	3340 (460-4320)	3325 (1230-4440)	0.52

The vaginal delivery group was younger than the CD group. PI values (MoM) were slightly higher in the vaginal delivery group in both exams, although this difference was not statistically significant for the first- and second-trimester uterine artery Doppler PI MoM values. Uterine artery PI index MoM values were not different between the cases with anterior placenta and posterior placenta (Table 2). In women with previous vaginal delivery and CS, no significant difference was found in the first- and second-trimester uterine artery PI MoM values by placenta location.

**Table 2:** Comparison of PAPP-A MoM values of uterine artery PI, birth weight, and gestational age at delivery by placenta location Median values are shown with a 95% confidence interval. *Chi-square test. PAPP-A: Pregnancy-associated plasma protein-A; PI: Pulsatility index; MoM: Multiples of the median.

	Anterior placenta (n = 108)	Posterior placenta (n = 101)	p-values
Vaginal delivery	50	52	p = 0.57*
Cesarean section	67	59	
PAPP-A	1.14 (1.05-1.23)	1.36 (1.16-1.49)	0.09
Uterine artery PI MoM in the first trimester	0.81 (0.77-0.88)	0.82 (0.77-0.89)	0.98
Uterine artery PI MoM in the second trimester	0.85 (0.81-0.9)	0.83 (0.78-0.88)	0.32
Gestational age at delivery	39 (38-39)	39 (38.69-39)	0.48
Birthweight	3305 (3214-3375)	3380 (3306-3473)	0.52

The intrauterine growth restriction rate was higher in the CD group (p = 0.001) (Table 3). Uterine artery notch was seen in 11 cases (10.7%) from the previous vaginal delivery group and eight cases (6.3%) from the previous CD group (p = 0.34) in the first-trimester exam. The persistence rate of uterine artery notch in the second-trimester exam was similar (p = 0.34).

**Table 3:** Comparison of pregnancy outcomes between women who had a vaginal delivery and those who had a cesarean delivery

	Vaginal delivery (n = 102)	Cesarean delivery (n = 126)	p-values
Premature rupture of membranes	3/102	2/126	0.81
Intrauterine growth restriction	0/102	5/121	0.001*
Small for gestational age	4/98	5/121	0.74
Oligohydramnios	1/101	0/126	0.91
Smoking	12/102	14/112	0.95
Preterm delivery	7/102	6/120	0.69
Persistent uterine artery notch	6/96	3/123	0.34

There was no difference in the placental location in the primiparous, previously c-sectioned group. In addition, there is no difference between the anterior and non-anterior placental groups in the first- and second-trimester PI MoM values.

## Discussion

A history of previous delivery by CS had been reported with increased risk for placenta accreta spectrum, placenta previa, preterm delivery, preeclampsia, low birth weight, stillbirth, and uterine rupture. Myometrial healing with scar tissue formation could affect trophoblast invasion and interaction with spiral arterioles. Recent data from human-animal studies suggest that maternal vascular changes are not limited to spiral arteries alone. The success of trophoblastic invasion, vascular changes in the arcuate and radial arteries, and formation of placental bed vascular anastomosis determine the flow pattern of uterine artery branches and PI values [10]. The PI values decrease longitudinally in singleton pregnancies in the forthcoming weeks. From the first trimester onwards, uterine artery PI was higher in the early preeclampsia group compared to the normal group. In the late preeclampsia group, uterine artery PI was significantly increased only after 33 weeks [11]. Parity was assessed as a confounding factor on uterine artery flow and Doppler indices.

Prefumo et al. reported that parity has a significant effect on uterine artery RI, and parous women had higher RI and a lower rate of diastolic notch in the second-trimester uterine Doppler studies [12]. Hafner et al. compared the PI and positivity of diastolic notch in the same pregnant women cohort in the first and second pregnancies at 22 weeks of gestation. They found that PI values did not differ significantly in the first and second pregnancies, whereas there was a small but marked difference in the PI in the right uterine artery compared to the left. Early diastolic notch was more frequently found in the first pregnancy [13]. Flo et al. compared uterine blood flow, RI, and PI in pregnant women with and without a previous CS. Although uterine artery volume blood flow was lower and uterine vascular resistance was higher, PI did not differ in pregnant women with a history of previous CS [14]. Another study by Filho et al. included 45 women in their second pregnancies and found that the uterine Doppler indices from 26 to 32 gestational weeks were not significantly different between women with prior c-sections and those with prior vaginal delivery [15]. Both studies confirm our results, and the availability of first-trimester uterine artery Doppler data, bigger sample size, and obstetrical follow-up data were the primary advantages of our study. In addition, Yapan et al. reported that the route of delivery in previous pregnancies did not influence any measurements or difference between the consecutive measurements of uterine artery Doppler indices during the pregnancy. Pregnancy outcomes were not different between the previous CD and vaginal delivery history except for the gestational age at delivery and the incidence of small for gestational age (SGA) [16]. Various studies indicate that a previous c-section is related to an increased risk of preeclampsia, antepartum hemorrhage, uterine rupture, intrauterine growth restriction (IUGR), preterm delivery, and stillbirth [17].

Torabi et al. observed an abnormally increased PI value in the second-trimester uterine artery Doppler in patients who had a previous CS and then preeclampsia compared to vaginal deliveries. They showed no statistically significant difference in separately adverse pregnancy outcomes such as oligohydramnios, IUGR, ablatio placenta, preterm delivery, and SGA; however, there is a statistically significant difference in total adverse pregnancy outcomes between vaginal and CS groups [18]. In addition, contrary to this study, we found that the IUGR rate was higher in the previous CD group. IUGR, as an adverse obstetric outcome, has been studied in literature as well, and a relationship has been shown between first-trimester PI value, PAPP-A MoM, and IUGR [19].

Placental location was another variable in our study. All CS procedures were performed by low transverse incision (Munro-Kerr incision). Some studies claim that the anterior-located placenta is more common in pregnancies with previous CS. Abnormal placental invasion on the anterior uterine wall might cause placental vascular resistance and elevated PI value in these groups. Our study compared PI between CS and vaginal delivery patients by placental location. According to our analysis, there is no PI difference between the previous vaginal delivery and CS group by placental location. We also found no difference in the adverse pregnancy outcomes according to placental location. Although Gómez et al. reported increased adverse obstetric outcomes and elevated PI values in pregnancies with the anterior-located placenta [4], we did not find a difference in PI values when the placenta lies on the anterior wall or other sites.

Uterine artery Doppler ultrasound examination was considered to be a safe and effective screening method for the early detection of possible obstetric complications. In many studies, persistent uterine artery notch has been linked to adverse obstetric outcomes. In our study, the positive uterine artery notch rate was similar between prior CS and vaginal delivery groups.

Nevertheless, our study has some limitations, such as the retrospective nature of the study and the limited sample size. For more accurate prediction and evaluation, more patient data is required. We tried to strengthen the patient data quality and eradicate the possible confounders by applying stringent inclusion and exclusion criteria. All cases were followed from the first trimester until delivery in our study. We only utilized the data from patients who were strictly followed by our maternal-fetal medicine unit and obstetrics clinic and were examined by the same specialist.

## Conclusions

Uterine artery Doppler scanning in early pregnancy is a safe and easy diagnostic method for detecting obstetric complications. Of course, more detailed and large-scale studies are needed. However, abnormally increased PI value in the second trimester should be followed closely regarding pregnancy complications. All pregnant women should be made aware of the second-trimester ultrasound scan.
